# Hypnotism

**Published:** 1901-10

**Authors:** 


Hypnotism. A Complete System of Method, Application and Use, Prepared for the Self-instruction of the Medical Profession. By L. W. De Laurence, Instructor at the School of Hypnotism and Suggestive Therapeutics, Pittsburg. Illustrated. Chicago: The Henneberry Co. 1901. [Price, $1.50.]

Works on hypnotism should emanate from professional men, and if they claim to be at all scientific, should be restricted to the profession. Treatises not intended for the profession, but for the laity should not receive the stamp of approval, in fact should be interdicted. It is a question whether the public at large should be allowed to understand the laws of hypnotism, to acquire the power to hypnotise, and to hypnotise indiscriminately. Much harm and injury has been done innocent individuals by unprincipled hypnotists, and the time has come when hypnotism should belong as exclusively to the medical profession as the right to prescribe poisonous drugs.

The book at hand caters more to the "intelligent person" than to the "medical man" and inasmuch as it goes quite deeply into the art of hypnotising, it should be restricted to the profession from the professional point of view. The author gives a clear, intelligible exposition of the laws and processes of hypnotism, taking up telepathy, magnetic healing, trance, dreams and allied psychological states.

Many illustrations of the different phases of hypnotism adorn the work. W. C. K.

